# Involvement of NEK2 and its interaction with NDC80 and CEP250 in hepatocellular carcinoma

**DOI:** 10.1186/s12920-020-00812-y

**Published:** 2020-10-27

**Authors:** Lu Zeng, Xiude Fan, Xiaoyun Wang, Huan Deng, Xiaoge Zhang, Kun Zhang, Shan He, Na Li, Qunying Han, Zhengwen Liu

**Affiliations:** 1 Department of Infectious Diseases, First Affiliated Hospital of Xi’an Jiaotong University, No. 277 Yanta West Road, Xi’an, 710061 Shaanxi Province People’s Republic of China; 2 Xi’an Medical University, Xi’an, 710021 Shaanxi Province People’s Republic of China

**Keywords:** NEK2, NDC80, CEP250, Hepatocellular carcinoma, Biomarker

## Abstract

**Background:**

NEK2 has an established involvement in hepatocellular carcinoma (HCC) but the roles of NEK2 and its interacting proteins in HCC have not been systematically explored.

**Methods:**

This study examined NEK2 and its interacting proteins in HCC based on multiple databases.

**Results:**

*NEK2* mRNA was highly expressed in HCC tissues compared with normal liver tissues. The survival of HCC patients with high *NEK2* mRNA expression was shorter than those with low expression. MAD1L1, CEP250, MAPK1, NDC80, PPP1CA, PPP1R2 and NEK11 were the interacting proteins of NEK2. Among them, NDC80 and CEP250 were the key interacting proteins of NEK2. Mitotic prometaphase may be the key pathway that NEK2 and its interacting proteins contributed to HCC pathogenesis. *NEK2*, *NDC80* and *CEP250* mRNAs were highly expressed in HCC tissues compared with normal liver tissues. The mRNA levels of *NEK2* were positively correlated with those of *NDC80* or *CEP250*. Univariate regression showed that *NEK2*, *NDC80* and *CEP250* mRNA expressions were significantly associated with HCC patients’ survival. Multivariate regression showed that *NDC80* mRNA expression was an independent predictor for HCC patients’ survival. Methylations and genetic alterations of *NEK2*, *NDC80* and *CEP250* were observed in HCC samples. The alterations of *NEK2*, *NDC80* and *CEP250* genes were co-occurrence. Patients with high mRNA expression and genetic alterations of *NEK2*, *NDC80* and *CEP250* had poor prognosis.

**Conclusions:**

NEK2 and its interacting proteins NDC80 and CEP250 play important roles in HCC development and progression and thus may be potentially used as biomarkers and therapeutic targets of HCC.

## Background

Hepatocellular carcinoma (HCC) has high morbidity and mortality and is the third leading cause of cancer-associated deaths worldwide [1]. Characteristics of HCC include aggressive, high malignancy, early metastasis and poor prognosis. With the application of alpha-fetoprotein (AFP) and improvement of imaging techniques such as ultrasound and computerized tomography and the progress of surgical and interventional therapy for HCC, the early diagnostic rate of HCC and the prognosis of HCC patients appear to have been increased and improved. However, many patients were diagnosed at the advanced stage of HCC and had poor prognosis. The 5-year survival rate in patients with early stage HCC is more than 70%, but it is less than 5% in patients with advanced HCC [2]. Therefore, the exploration of biomarkers for the early diagnosis and targets for the therapy of HCC is an important research focus.

Never in mitosis gene A-related kinase 2 (NEK2) belongs to serine/threonine kinase and widely exists in the centrosome of cells [3]. NEK2 plays important roles in regulating the mitosis process, including chromatin separation, spindle assembly, centrosome division and tumor drug resistance [4–6]. Studies have shown that the high mRNA expression of *NEK2* was related to the incidence, differentiation, metastasis and prognosis of multiple myeloma [7], pancreatic ductal adenocarcinoma [8], ovarian cancer [9], colorectal cancer [10] and breast cancer [11]. Studies also showed the involvement of NEK2 in the development and progression of HCC [12] and overexpression of *NEK2* was indicated to be associated with a poor survival in HCC patients [13]. However, most of the previous studies were conducted in small sample sizes of patients and the possible network proteins of NEK2 remain to be investigated. Therefore, this study systematically examined the role of NEK2 in HCC, mined the NEK2 interacting proteins and explored the underlying mechanisms by the analyses of multiple databases to provide evidence for the potential of NEK2 and its interacting proteins in the diagnosis and therapy of HCC.

## Methods

Multiple databases were used to systematically investigate the potential involvement of NEK2 and its interacting proteins in HCC and explored the possible mechanisms. First, the mRNA expression of *NEK2* in HCC and the association of *NEK2* mRNA expression levels with prognosis of HCC patients were analyzed by UALCAN. Second, the upstream or downstream interacting proteins of NEK2 were mined using GeneSense and the relationships between NEK2 and its interacting proteins and the key interacting proteins were verified and identified by STRING (Search Tool for the Retrieval of Interacting Genes/Proteins). Third, the functional enrichment pathways of NEK2 and its interacting proteins were explored by DAVID (the Database for Annotation, Visualization and Integrated Discovery) and KOBAS (KEGG Orthology Based Annotation System) and the pathways of NEK2 and its interacting proteins were verified using Reactome. Fourth, the effects of NEK2 and its key interacting proteins on HCC patients’ survival were examined using data downloaded from TCGA (The Cancer Genome Atlas) and the mRNA expression of *NEK2*, *NDC80* and *CEP250* in HCC were analyzed by Oncomine. Lastly, the genetic alterations of *NEK2* and the genes of NEK2 key interacting proteins in HCC, the relationship of the genetic alterations between the genes, and the influence of the genetic alterations on HCC patients’ survival were analyzed by cBioPortal. The flow chart of the study was shown in Additional file 1: Figure S1.

### UALCAN database

UALCAN (https://ualcan.path.uab.edu/analysis.html) is a user-friendly and interactive website, which provides easy access to publicly available cancer transcriptome data (TCGA and MET500 transcriptome sequencing). Plots depicting gene expression and patient survival information based on different gene expression can be obtained by UALCAN. This study explored the mRNA expression of *NEK2* in HCC in comparison with normal liver, and plotted the survival curve of HCC patients with different mRNA expression levels of *NEK2* by UALCAN.

### GeneSense database

GeneSense (https://www.biomedsense.org/genesense.php) is a web-based platform that allows users to construct, visualize, manipulate and analyze gene information. It aims to assist researchers to find the optimal gene regulatory factors by associated networks. GeneSense adopts a number of methods to annotate protein–protein interaction (PPI). The PPI network which GeneSense can construct has three styles, namely nodenet, leafnet and loopnet. Nodenet supports basic queries of PPI network for upstream or downstream protein analysis. Leafnet model visualizes the complexity of the queried protein and its upstream or downstream proteins. Loopnet visualizes the upstream and downstream target of the queried protein. This study used loopnet style to visualize the possible upstream and downstream interacting proteins of NEK2 and both the upstream and downstream proteins were all considered to be NEK2 interacting proteins.

### STRING: Protein–protein interaction network construction

STRING: functional protein association networks (https://string-db.org/), is a website which can predict PPIs. We used STRING to verify PPI network of NEK2 and its interacting proteins obtained from GeneSense*.* An interaction score > 0.9 was regarded as high confidence PPIs and the related interacting protein was considered to be a key interacting protein of NEK2.

### DAVID and KOBAS functional enrichment analyses

The functional and signaling pathway analyses of NEK2 and its interacting proteins were performed on two public database platforms, DAVID (https://david.ncifcrf.gov/), and KOBAS 3.0 (https://kobas.cbi.pku.edu.cn/index.php). DAVID database was used to acquire the Gene Ontology (GO) terms that include three categories, namely molecular function (MF), cellular component (CC) and biological process (BP). KOBAS 3.0 database was used to obtain the main Kyoto Encyclopedia of Genes and Genomes (KEGG) pathways. The enriched GO terms and KEGG pathways of NEK2 and its interacting proteins were visualized by R software and Cytoscape 3.6.1.

### Reactome database

Reactome database (https://reactome.org/what-is-reactome) provides intuitive bioinformatics tools for the visualization, interpretation and analysis of pathway knowledge, and can present data which describe possible pathways if annotated proteins and small molecules were present in a cell. The possible pathways that interesting proteins can be obtained and the details of the pathways can be visualized. This study used the ReactomeFIPlugIn in Cytoscape software to analyze the pathways that NEK2 and its interacting proteins may involve in and to acquire the pathway diagram.

### TCGA database

The Cancer Genome Atlas (TCGA) database (https://cancergenome.nih.gov/), which integrates gene expression data and clinical data, was used to verify and analyze the prognostic value of NEK2 and its interacting proteins in HCC patients. R “survival” package [14] was used to visualize the results. The clinical covariates of 373 HCC patients from TCGA database were included in Cox proportional hazard model to identify the independent predictor for HCC patients’ survival. The correlations of mRNA expression between *NEK2* and *NDC80* or *CEP250* were analyzed.

### Oncomine database

Oncomine database (https://www.oncomine.org/resource/login.html), an online microarray database, was used to explore and confirm the mRNA expression levels of *NEK2* and the genes of NEK2 key interacting proteins in HCC. The conditions for filter setting were as follows: Gene: “NEK2 or its key interacting proteins”, Analysis Type: “Cancer vs. Normal Analysis”, Cancer Type: “Hepatocellular Carcinoma”, Data Type: “mRNA”, Sample Type: “Clinical Specimen”. The studies incorporating mRNA expression data of *NEK2* and the genes of NEK2 key interacting proteins were obtained, and ordered by “Over-expression: Gene Rank”. The different studies could be compared by conducting meta-analysis to demonstrate the reliability of these data.

### cBioPortal database

cBioPortal database (https://www.cbioportal.org/) provides visualization tools for research and analysis of cancer gene data. The integrated genomic data types include somatic cell mutation, DNA copy number changes, mRNA and microRNA expression, DNA methylation, protein abundance and phosphoprotein abundance. The genetic alterations, relationship between genetic alterations, methylation, co-expression and the survival of HCC patients in relation to NEK2 and its key interacting proteins were explored through the database. “Liver Hepatocellular Carcinoma (TCGA, Provisional)” study was selected for analyzing, and there were 366 patients/samples with mutation and copy number alteration (CNA) data which comprise “mutation, putative copy-number alterations” from GISTIC, and mRNA expression z-Scores (RNA Seq V2 RSEM)” information. The mRNA expression z-Score threshold was ± 2.0.

### Statistical analysis

Statistical analyses were carried out by SPSS statistical software (Version 22.0; Inc., IBM, NY, USA) and Graghpad Prism software (Version 6.0; Inc., Graghpad, San Diego, CA, USA). The mRNA expression was expressed as mean ± standard deviation (SD), and the expression differences between groups were compared by nonparametric test. Cox regression model was used to identify independent predictors for HCC patients’ survival. Survival of HCC patients was estimated using the Kaplan–Meier method and compared by the log-rank test. The mRNA expression correlation between *NEK2* and other genes were evaluated by Spearman correlation analyses. A *p* value of < 0.05 was considered statistically significant.

## Results

### mRNA expression of *NEK2* in normal and HCC tissues and its effect on HCC patient survival by UALCAN

**Fig. 1:**
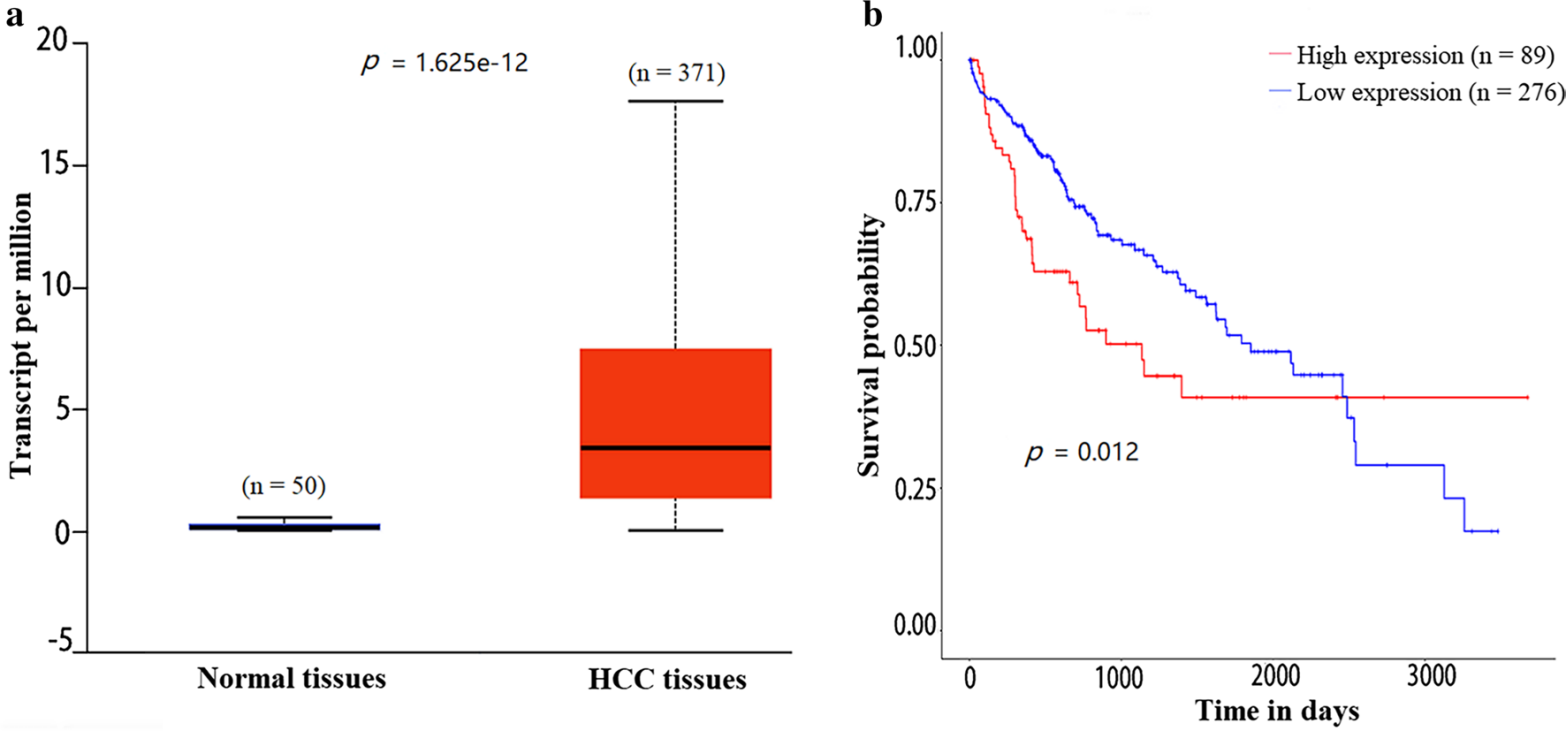
The mRNA expression of *NEK2* in normal tissues and HCC tissues (**a**) and the effect of *NEK2* mRNA expression on HCC patients' survival (**b**) by UALCAN analysis

Based on UALCAN analyses [15], the mRNA expression of *NEK2* was elevated in HCC tissues (n = 371) compared with normal liver tissues (n = 50, *p* = 1.625 × 10^–12^, Fig. 1a). Survival analysis of patients with different levels of *NEK2* mRNA expression demonstrated that the survival time of patients with high *NEK2* mRNA expression (n = 89) was shorter than those with low expression (n = 276, *p* = 0.012, Fig. 1b).

### The upstream and downstream interacting proteins of NEK2 by Genesense

**Fig. 2:**
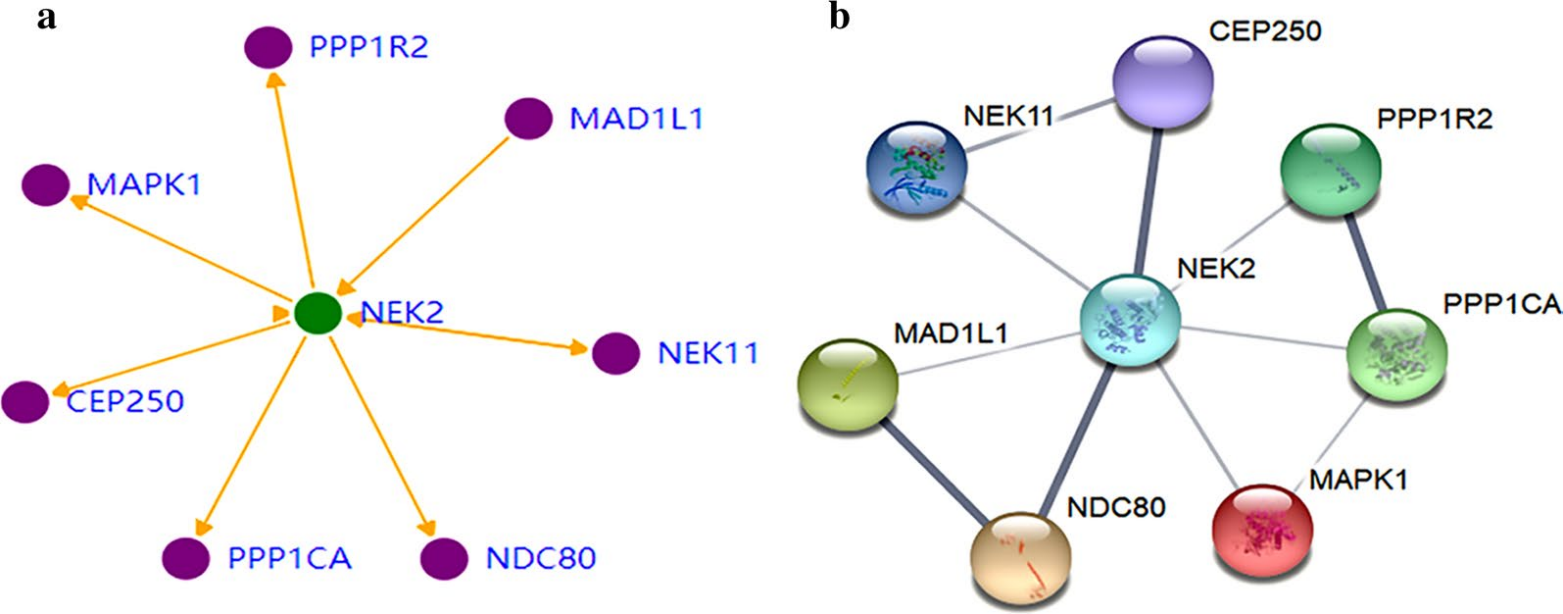
The upstream and downstream interacting proteins of NEK2 by Genesense analysis and the protein–protein interaction network of NEK2 and its interacting proteins by STRING database. **a** The upstream and downstream interacting proteins of NEK2 by Genesense analysis. The protein with an arrow pointing to NEK2 is the upstream protein of NEK2, and the protein with an arrow pointing away from NEK2 is the downstream protein of NEK2. **b** The protein–protein interaction network of NEK2 and its interacting proteins by STRING database. The line between proteins indicates that proteins interact with each other, and the thickness of the line indicates the interaction score

The upstream and downstream interacting proteins of NEK2 were mined by Genesense website [16]. MAD1L1 [MAD1 mitotic arrest deficient-like 1 (yeast)] [17] was found to be the upstream protein of NEK2 while CEP250 (centrosomal protein 250 kDa) [18], MAPK1 (mitogen-activated protein kinase 1) [19], NDC80 (nuclear division cycle 80) [20], PPP1R2 [protein phosphatase 1, regulatory (inhibitor) subunit 2] [21] and PPP1CA (protein phosphatase 1 catalytic subunit alpha) were the downstream proteins of NEK2. NEK11 [NIMA (never in mitosis gene a)- related kinase 11] [22] was indicated to be both the upstream and downstream protein of NEK2 (Fig. 2a).

### Protein–protein interaction by STRING database

The PPI network was constructed by STRING database [23] to verify the association between NEK2 and its interacting proteins. The results showed that NEK2 had strong interaction with its downstream and upstream proteins. The PPI network’s enrichment *p*-value was 1.23e−07. Among these proteins, NDC80 [24, 25] and CEP250 [26] had the highest interaction scores (Fig. 2b). The interaction scores between NEK2 and NDC80, and NEK2 and CEP250 were 0.994 and 0.995, respectively, indicating that NDC80 and CEP250 were NEK2 key interacting proteins. The main GO and KEGG pathways involving NEK2 and its interacting proteins analyzed by STRING database were downloaded (Table 1). Cell cycle [false discovery rate (FDR) = 2.78E−05], regulation of mitotic cell cycle phase transition (FDR = 4.01E−05) and mitotic cell cycle process (FDR = 0.0002) were the main GO biological progress pathways. Phosphatase binding (FDR = 0.0026), protein serine/threonine kinase activity (FDR = 0.0219) and protein phosphatase binding (FDR = 0.0265) were the main GO molecular function pathways. Microtubule organizing center (FDR = 0.00037), centrosome (FDR = 0.00059) and condensed chromosome kinetochore (FDR = 0.00059) were the main GO cellular component pathways. Long-term potentiation (FDR = 0.0337), progesterone-mediated oocyte maturation (FDR = 0.0356) and cGMP-PKG signaling pathway (FDR = 0.0358) were the main KEGG pathways. The proteins enriched in each pathways were displayed in Table 1.

**Table 1:** The main GO and KEGG pathways involving NEK2 and its interacting proteins downloaded from STRING database

Categories	Pathway ID	Pathway description	Gene count	FDR	Genes
Biological process (GO)	GO:0007049	Cell cycle	7	2.78E-05	*CEP250*,*MAD1L1*,*MAPK1*,*NDC80*,*NEK11*,*NEK2*,*PPP1CA*
	GO:1901990	Regulation of mitotic cell cycle phase transition	5	4.01E-05	*CEP250*,*MAD1L1*,*NDC80*,*NEK11*,*NEK2*
	GO:1903047	Mitotic cell cycle process	5	0.00021	*CEP250*,*MAD1L1*,*NDC80*,*NEK11*,*NEK2*
Molecular function (GO)	GO:0019902	Phosphatase binding	3	0.0026	*MAPK1*,*NEK2*,*PPP1CA*
	GO:0004674	Protein serine/threonine kinase activity	3	0.0219	*MAPK1*,*NEK11*,*NEK2*
	GO:0019903	Protein phosphatase binding	2	0.0265	*NEK2*,*PPP1CA*
Cellular component (GO)	GO:0005815	Microtubule organizing center	5	0.00037	*CEP250*,*MAD1L1*,*MAPK1*,*NDC80*,*NEK2*
	GO:0005813	Centrosome	4	0.00059	*CEP250*,*MAD1L1*,*NDC80*,*NEK2*
	GO:0000777	Condensed chromosome kinetochore	3	0.00059	*MAD1L1*,*NDC80*,*NEK2*
KEGG pathways	hsa04720	Long-term potentiation	2	0.0337	*MAPK1*,*PPP1CA*
	hsa04914	Progesterone-mediated oocyte maturation	2	0.0356	*MAD1L1*,*MAPK1*
	hsa04022	cGMP-PKG signaling pathway	2	0.0358	*MAPK1*,*PPP1CA*

*FDR* false discovery rate

### Functional enrichment by DAVID and KOBAS 3.0 databases

**Fig. 3:**
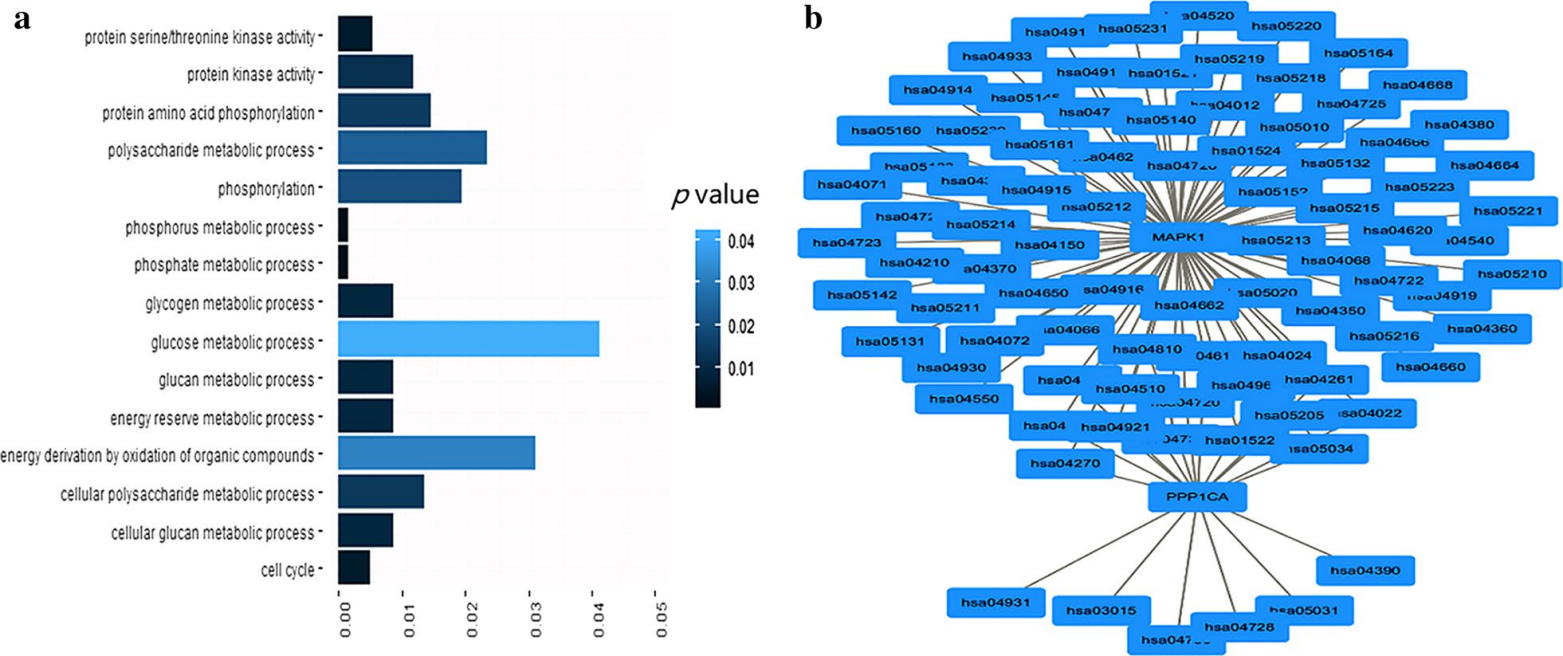
The main GO terms that NEK2 and its interacting proteins enriched and the main KEGG pathways that NEK2 and its interacting proteins enriched. **a** The main GO terms that NEK2 and its interacting proteins enriched. The shorter the bar graph and the darker the color of each term, the greater the statistical difference is. **b** The main KEGG pathways that NEK2 and its interacting proteins enriched. MAPK1 and PPP1CA enriched the most KEGG pathways

In order to further analyze the functional enrichment of NEK2 and its interacting proteins (MAD1L1, CEP250, MAPK1, NDC80, NEK11, PPP1CA and PPP1R2) in HCC, GO and KEGG pathways were investigated based on DAVID [27] and KOBAS 3.0 [28] databases. The details of the main GO terms and KEGG pathways were shown in Table 2, and the main GO terms and KEGG pathways were demonstrated in Fig. 3a and Fig. 3b, respectively. Phosphorus metabolic process (*p* = 0.001496693), phosphate metabolic process (*p* = 0.001496693), and cell cycle (*p* = 0.005071148) were significantly enriched in the category of GO biological progress. Protein serine/threonine kinase activity (*p* = 0.005262684) was the mainly enriched pathway in the category of GO molecular function. There were no pathways enriched in GO cellular component. Long-term potentiation (*p* = 0.000150473), oocyte meiosis (*p* = 0.000484062), vascular smooth muscle contraction (*p* = 0.000508059), platelet activation (*p* = 0.000524374) and insulin signaling pathway (*p* = 0.000663998) were found to be the significantly enriched KEGG pathways (Table 2). MAPK1 and PPP1CA were the major proteins involved in the KEGG pathways (Fig. 3b).

**Table 2:** The main GO and KEGG pathways associated with NEK2 and its interacting proteins by DAVID and KOBAS 3.0 databases analyses

Categories	Pathway ID	Pathway description	Gene count	*p*-value	Genes
Biological process (GO)	GO:0006793	Phosphorus metabolic process	4	0.0015	*MAPK1*, *PPP1CA*, *NEK2*, *NEK11*
	GO:0006796	Phosphate metabolic process	4	0.0015	*MAPK1*, *PPP1CA*, *NEK2*, *NEK11*
	GO:0007049	Cell Cycle	3	0.00507	*MAPK1*, *PPP1CA*, *NEK2*
Molecular function (GO)	GO:0004674	Protein serine/threonine kinase activity	3	0.00526	*MAPK1*, *NEK2*, *NEK11*
KEGG pathways	hsa04720	Long-term potentiation	2	0.00015	*MAPK1*, *PPP1CA*
	hsa04114	Oocyte meiosis	2	0.00048	*MAPK1*, *PPP1CA*
	hsa04270	Vascular smooth muscle contraction	2	0.00051	*MAPK1*, *PPP1CA*
	hsa04611	Platelet activation	2	0.00052	*MAPK1*, *PPP1CA*
	hsa04910	Insulin signaling pathway	2	0.00066	*MAPK1*, *PPP1CA*

### Reactome analyses

Reactome dababase [29] was used to verify the pathways that NEK2 and its interacting proteins were likely to participate in. The Analyze Data tool of Reactome was used to perform the analyses. NEK2 and its interacting proteins list were inputs. Human was chosen as species. Results showed that there were 122 significant pathways which NEK2 and its interacting proteins involved in (*p* < 0.05) (Additional file 2: Figure S2). The top 5 pathways were displayed in Table 3. Among the results, mitotic prometaphase (*p* = 2.63E−06), M phase (*p* = 2.24E−05), and cell cycle, mitotic (*p* = 9.11E−05) were the three pathways including the most queried genes. The details of mitotic prometaphase pathway were shown in Additional file 3: Figure S3. The main biological processes that NEK2 and its interacting proteins involved in included recruitment of NuMA (nuclear mitotic apparatus protein) to mitotic centrosomes, kinetochore capture of astral microtubules, resolution of sister chromatid cohesion and condensation of prometaphase chromosomes.

**Table 3:** The top 5 pathways associated with NEK2 and its interacting proteins by Reactome analyses

Pathway description	Gene count	*p* value	FDR	Genes
Mitotic prometaphase	4	2.63E−06	3.52E-04	*CEP250*,*NEK2*,*MAD1L1*,*NDC80*
M phase	4	2.24E−05	1.50E-03	*CEP250*,*NEK2*,*MAD1L1*,*NDC80*
Cell cycle, mitotic	4	9.11E−05	4.01E-03	*CEP250*,*NEK2*,*MAD1L1*,*NDC80*
RHO GTPase effectors	3	3.20E−04	0.0106	*MAPK1*,*MAD1L1*,*NDC80*
Opioid signaling	2	7.85E-−04	0.0123	*MAPK1*,*PPP1CA*

*FDR* false discovery rate

### Survival analyses by TCGA database

**Fig. 4:**
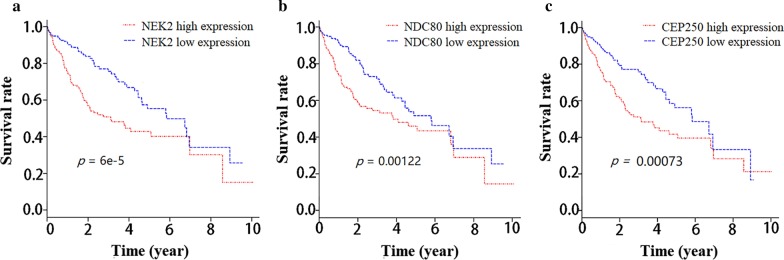
Effect of different *NEK2* (**a**), *NDC80* (**b**) and *CEP250* (**c**) mRNA expression levels on HCC patients' survival by TCGA database

Clinical data of HCC patients were downloaded from TCGA database [30] and the effects of different mRNA expression levels of *NEK2* and the genes of NEK2 interacting proteins on prognosis of HCC patients were investigated. A total of 373 HCC patients’ clinical data from TCGA database were downloaded, and the patients were divided into high mRNA expression group (n = 187) and low mRNA expression group (n = 186) based on the median mRNA expression value of interesting genes. Only NEK2, NDC80 and CEP250 were found to be associated with the survival of HCC patients (*P* = 6 × 10^–5^, *P* = 0.00122 and *P* = 0.00073, respectively, Fig. 4). The detailed information of 373 HCC patients from TCGA database were displayed in Additional file 4: Table S1. The *NEK2*, *NDC80* and *CEP250* mRNA expressions were different in relation to age, weight, recurrence status, histological grade, family HCC history, pathologic stage, tumor size, and survival time. Cox models incorporating clinical covariates of the 373 HCC patients from TCGA database were set up. Clinical covariates and mRNA expression values of the three genes (*NEK2*, *NDC80*, *CEP250*) were fitted into Cox regression models (Table 4). In univariate Cox regression analysis, HCC recurrence, pathologic stage, tumor size, and *NEK2*, *NDC80* and *CEP250* mRNA expression were all significantly associated with HCC patients’ survival. In multivariate Cox regression models (the covariates with *p* < 0.2 in univariate analysis were included), age, pathologic stage, and *NDC80* mRNA expression were independent predictors for HCC patients’ survival. The correlation between *NDC80*, *CEP250* and *NEK2* mRNA expression were analyzed. Reactome analysis revealed that CCNB1, CCNB2, and CDK1 were involved in mitotic prometaphase pathway (Additional file 3: Figure S3). Therefore, *CCNB1*, *CCNB2*, and *CDK1* were also included in correlation analysis. The mRNA expressions of all the five genes (*NDC80*, *CEP250*, *CCNB1*, *CCNB2* and *CDK1*) were positively correlated with *NEK2* mRNA expression (Additional file 5: Figure S4).

**Table 4:** Cox regression model of clinical covariates and the NEK2, NDC80 and CEP250 mRNA expression for HCC patients’ survival

	Univariate analysis			Multivariate analysis		
	HR	95% CI	*p* value	HR	95% CI	*p* value
Age	1.012	0.998–1.026	0.091	1.016	1.000–1.032	0.045
Gender (ref. Female)	1.260	0.884–1.795	0.201			
Family history (ref. No)	0.828	0.574–1.195	0.314			
Recurrence (ref. No)	0.587	0.371–0.926	0.022			
Weight	0.993	0.984–1.003	0.195			
Platelet count	1.000	1.000–1.000	0.696			
Child–Pugh score (ref. A)						
B	0.469	0.064–3.408	0.454			
C	0.747	0.092–6.055	0.785			
Histological grade (ref.G1)						
G2	0.595	0.220–1.609	0.306			
G3	0.692	0.277–1.731	0.431			
G4	0.705	0.278–1.786	0.461			
Pathologic stage (ref.Stage I)						
Stage II	0.178	0.055–0.577	0.004	1.240	0.735–2.093	0.420
Stage III	0.254	0.077–0.842	0.025	2.438	1.562–3.805	2.438
Stage IV	0.492	0.152–1.590	0.236	7.033	2.152–22.979	0.001
Primary tumor range (ref.T1)						
T2	0.186	0.093–0.372	< 0.001			
T3	0.267	0.129–0.550	< 0.001			
T4	0.497	0.248–0.997	0.049			
NEK2 mRNA expression	1.001	1.000–1.001	0.001			
NDC80 mRNA expression	1.001	1.001–1.002	< 0.001	1.001	1.001–1.002	< 0.001
CEP250 mRNA expression	1.000	1.000–1.001	0.027			

*HR* hazard ratio, *CI* confidence interval, *Ref* reference

### The mRNA expression of *NEK2* and the genes of NEK2 key interacting proteins (NDC80 and CEP250) by Oncomine database

**Fig. 5:**
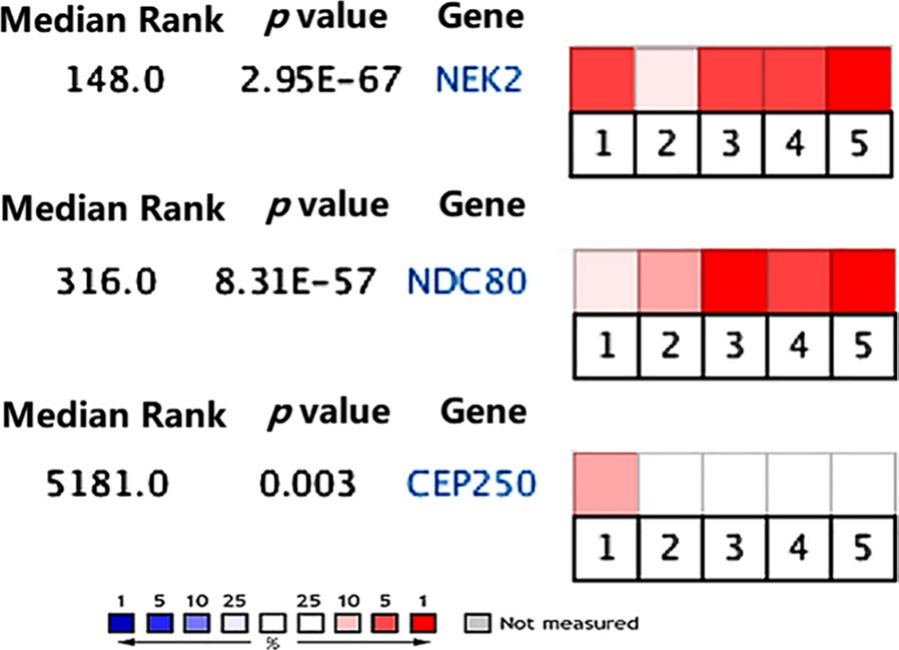
Meta-analysis of the mRNA expression levels of *NEK2*, *NDC80* and *CEP250* in five HCC vs. normal liver studies. The rank of a gene is the median rank for that gene across each of the analyses. The *p* value for a gene is its *p* value for the median-ranked analysis. Red represents high mRNA expression level, and blue represents low mRNA expression level. The darker the color, the more obvious the difference. The five studies were *Chen Liver* (1), *Mas Liver* (2), *Roessler Liver* (3), *Roessler Liver 2* (4), and *Wurmbach Liver* (5)

The mRNA expression of *NEK2* and the genes of NEK2 key interacting proteins (NDC80 and CEP250) in HCC was analyzed by Oncomine database [31]. There were five eligible HCC studies: Chen Liver [32], Mas Liver [33], Roessler Liver [34], Roessler Liver 2 [34] and Wurmbach Liver [35]. The detailed information was Chen Liver: 99 HCC samples and 77 normal samples, Mas Liver: 38 HCC samples and 19 normal liver samples, Roessler Liver: 22 HCC samples and 21 normal samples, Roessler Liver 2: 225 HCC samples and 220 normal samples, and Wurmbach Liver: 35 HCC samples and 10 normal samples. Meta-analysis of the mRNA expression level of the three genes in the five studies showed that *NEK2* (*p* = 2.95 × 10^–67^), *NDC80* (*p* = 8.31 × 10^–57^) and *CEP250* (*p* = 0.003) were highly expressed in HCC (Fig. 5). *NEK2* was obviously over-expression in 4 studies, especially in Wurmbach Liver study. *NDC80* was also obviously over-expression in 4 studies, especially in Roessler Liver and Wurmbach Liver studies. *CEP250* was only obviously over-expression in Chen Liver study. None of the the three genes showed low mRNA expression in the studies.

### Genetic alteration and mutual exclusivity of *NEK2*, *NDC80* and *CEP250*, and the effects on the survival of HCC patients by cBioPortal database

**Fig. 6:**
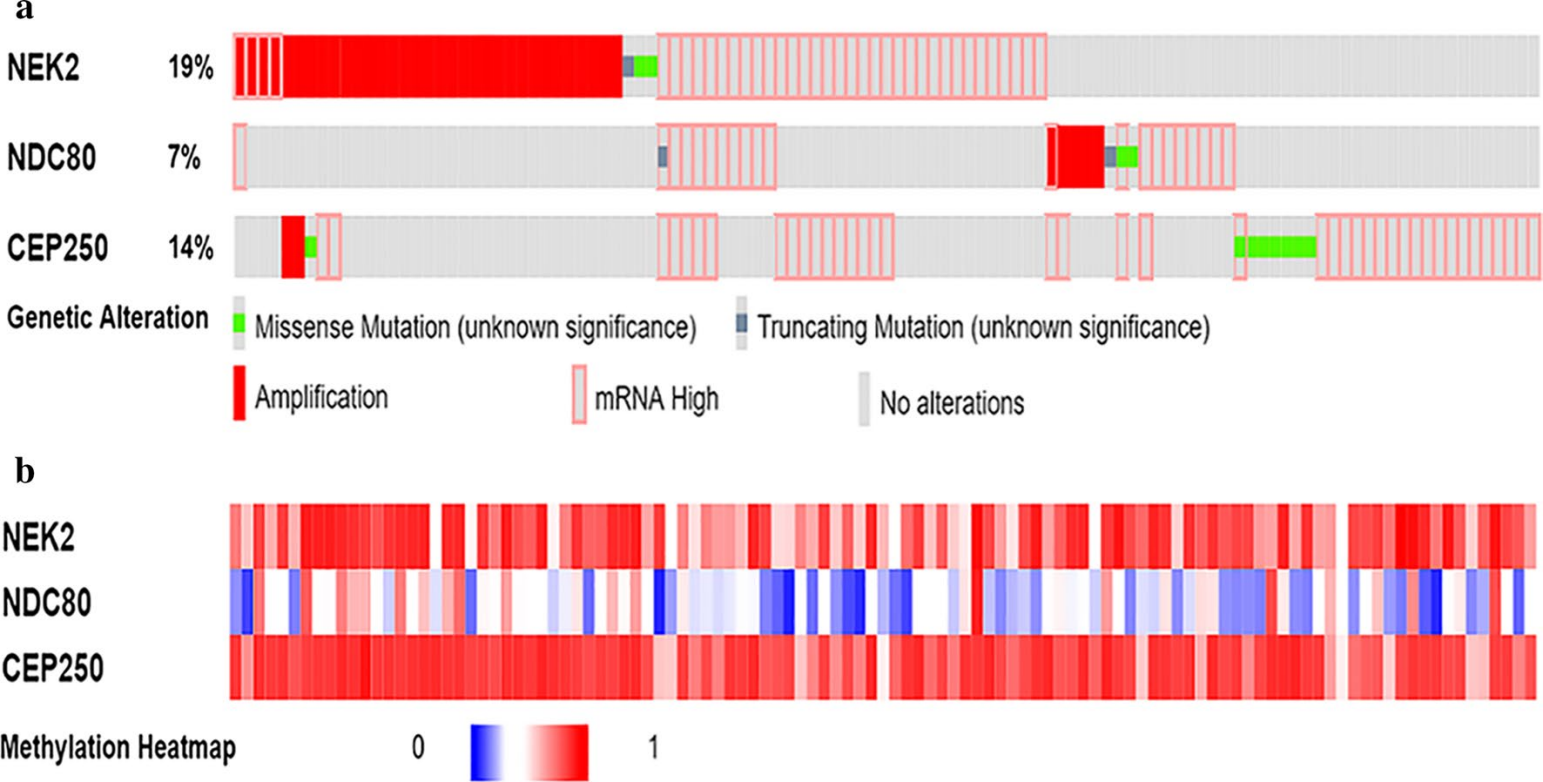
The genetic alterations and methylation level of *NEK2*, *NDC80* and *CEP250* in HCC. **a** The genetic alterations of *NEK2*, *NDC80* and *CEP250* in HCC. The queried genes (*NEK2*, *NDC80*, *CEP250*) were altered in 111/366 (30%) of queried samples. The genetic alteration frequencies of *NEK2*, *NDC80* and *CEP250* were 19%, 7% and 14%, respectively. The detailed genetic alteration types included Amplification, Missense Mutation, Truncating Mutation and mRNA High. **b** The methylation level of *NEK2*, *NDC80* and *CEP250* in HCC. Red represents high methylation level, and blue represents low methylation level

**Fig. 7:**
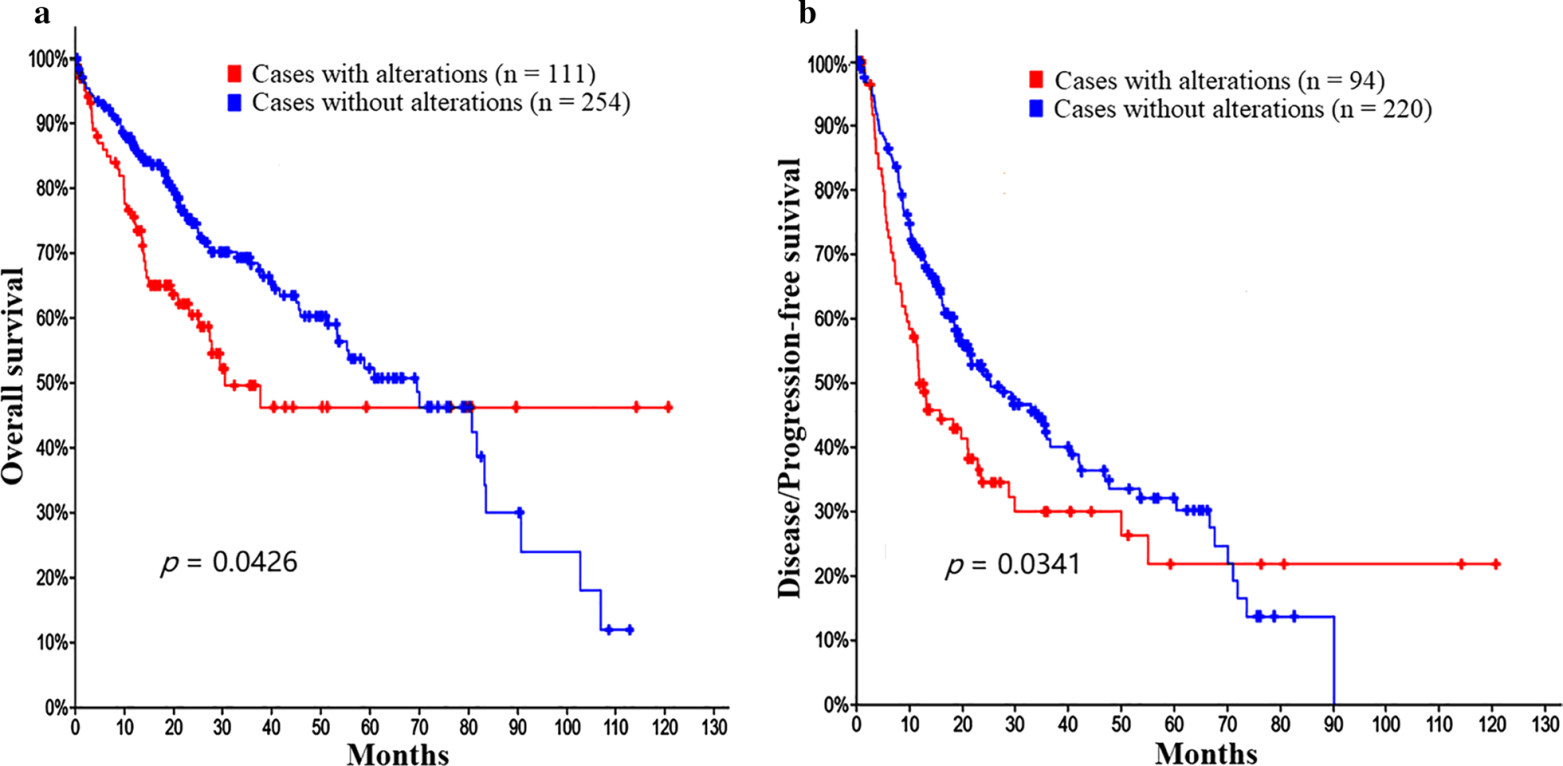
Effects of *NEK2*, *NDC80* and *CEP250* genetic alterations on overall survival (**a**) and disease/progression-free survival (**b**) of HCC patients by cBioPortal database analyses

Oncoprint feature of the cBioPortal database [36] was used to determine the genetic alteration frequency and epigenetic alteration level of *NEK2*, *NDC80* and *CEP250* in HCC. The queried genes (*NEK2*, *NDC80*, *CEP250*) were altered in 111/366 (30%) of queried samples. The genetic alteration frequencies of *NEK2*, *NDC80* and *CEP250* were 19%, 7% and 14%, respectively. Among these, *NEK2* has the highest frequencies of gene mutation and copy-number alterations. The detailed genetic alteration types included amplification, missense mutation, truncating mutation and mRNA high, although missense mutation and truncating mutation remained unknown significance (Fig. 6a). For epigenetic alterations, the methylation level of *NEK2* and *CEP250* were particularly significant in these samples (Fig. 6b). The mutual exclusivity function of cBioPortal database was used to determine whether there were any relationship between the genetic alterations of *NEK2*, *NDC80* and *CEP250*. The results showed that the relationships between the alterations of the three genes were all co-occurrence (Table 5). The correlation coefficient between *NEK2* and *NDC80* or *CEP250* mRNA expression were 0.87 and 0.59, respectively (Additional file 5: Figure S5). The overall survival and disease/progression-free survival rates for HCC patients with *NEK2*, *NDC80* and *CEP250* genetic alterations were significantly lower than those without the alterations of the three gene (*p* = 0.0426 and 0.0341, respectively, Fig. 7).

**Table 5:** Relationships between the genetic alterations of *NEK2*, *NDC80* and *CEP250*

Gene A	Gene B	Log odds ratio	*p* value	Adjusted *p* value	Tendency
*NEK2*	*CEP250*	1.267	< 0.001	< 0.001	Co-occurrence
*NEK2*	*NDC80*	1.182	0.006	0.017	Co-occurrence
*NDC80*	*CEP250*	1.27	0.006	0.018	Co-occurrence

## Discussion

Identification of the differences and mechanisms of genes involved in HCC are of great significance for biomarkers and therapeutic targets. NEK2 is a microtubule-binding protein that regulates spindles in human cells. Overexpression of *NEK2* mRNA can lead to chromosomal instability, abnormal cell proliferation and drug resistance [13]. Studies showed that *NEK2* mRNA expression was abnormal in HCC. A study in 63 cases of HCC and matched adjacent non-tumorous liver tissues showed that the mRNA expression of *NEK2* was increased, and compared to patients with low mRNA expression, patients with high *NEK2* mRNA expression had a poor prognosis. Possible mechanism was that NEK2 influenced the invasion and metastasis of HCC by activating AKT signaling pathway and promoting the expression of matrix metalloproteinases-2 (MMP-2) protein [37]. Another study also demonstrated that the prognosis of HCC patients with high mRNA expression of *NEK2* were poorer than patients with low expression [38]. However, study has shown that the mRNA expression of *NEK2* in HCC tissues was lower than adjacent normal tissues, and was associated with larger tumor diameter, higher alpha-fetoprotein (AFP) concentration, higher tumor stage, worse prognosis and shorter survival time [39]. The differences between the studies need to be clarified by more studies.

In the present study, the mRNA expression of *NEK2* in HCC was systematically examined by multiple databases. Results showed that *NEK2* mRNA was highly expressed in HCC, and HCC patients with high *NEK2* mRNA expression had poor prognosis. MAD1L1, CEP250, MAPK1, NDC80, PPP1CA, PPP1R2 and NEK11 were found to be the possible upstream or downstream interacting proteins of NEK2. Among these proteins, MAD1L1 was the upstream protein and CEP250, MAPK1, NDC80, NEK11, PPP1CA and PPP1R2 were the downstream proteins of NEK2. NEK11 was both the upstream and downstream protein of NEK2. PPI network confirmed the relationship between NEK2 and its interacting proteins, and NDC80 and CEP250 were found to be NEK2 key interacting proteins. STRING database displayed some GO terms and KEGG pathways that NEK2 and its interacting proteins participated in. Functional pathways by DAVID and KOBAS 3.0 databases also showed that NEK2 and its interacting proteins were involved in important biological processes. Of note, the analysis results of the databases had some differences. To increase the reliability of the results, Reactome database was chosen for further verification and the results showed that NEK2, NDC80, CEP250, MAPK1 and PPP1CA all participated in important biological pathways. Cell cycle was consistently presented in all the three pathway analyzing tools and mitotic prometaphase, the subset of cell cycle, was the most significant pathway which NEK2 and its interacting proteins involved in. Further analysis indicated that mRNA expressions of the three genes (CCNB1, CCNB2 and CDK1) involved in mitotic prometaphase all had high correlation coefficient with NEK2 mRNA expression. We infer that there may be some interrelations between NEK2 and CCNB1, CCNB2 or CDK1, indirectly suggesting the role of NEK2 in mitotic prometaphase pathway. Kaplan–Meier survival analyses of NEK2 and its interacting proteins in HCC data downloaded from TCGA database showed that only NEK2, NDC80 and CEP250 had significant influence on the prognosis of HCC patients. Taken together, NEK2, NDC80 and CEP250 might play important roles in the pathogenesis of HCC.

Oncomine database analyses showed that *NEK2*, *NDC80* and *CEP250* mRNA were over-expression in HCC. cBioPortal database analyses indicated that methylations, genetic and epigenetic alterations of *NEK2*, *NDC80* and *CEP250* were found in HCC samples. The survival time of HCC patients with *NEK2*, *NDC80* and *CEP250* genetic alterations were significantly shorter than those without. In univariate Cox regression analyses, *NEK2*, *NDC80* and *CEP250* mRNA expression were all significantly related to HCC patients’ survival. In multivariate Cox regression model, only NDC80 was the independent predictor of HCC prognosis. These outcomes indicated the critical role of NDC80 in HCC. cBioPortal database revealed the alterations of the three genes were co-occurrence, and clinical correlation analysis also showed the mRNA expression of *NDC80*, *CEP250* and *NEK2* were positively correlated. Genensense demonstrated that NDC80 and CEP250 were the downstream protein of NEK2. Therefore, NEK2 may play its role in HCC through interaction with NDC80 and CEP250, especially NDC80. Of course, studies are required to confirm this hypothesis.

NDC80 acts as a component of the essential kinetochore-associated NDC80 complex, plays a role in chromosome congression and is essential for the end-on attachment of the kinetochores to spindle microtubules. *NDC80* mRNA expression levels in HCC tissues were found to be significantly higher than those in the adjacent tissues, and NDC80 was believed to contribute to HCC progression by reducing apoptosis and overcoming cell cycle arrest [40]. *NDC80* silencing was shown to significantly reduce Hep3 B cell proliferation and colony formation [41]. *CEP250* encodes a core centrosomal protein contributed to the centrosome cohesion, centriole biogenesis, and centrosome duplication processes at different cell cycle stages. Phosphorylation of the C-terminal region of *CEP250* by NEK2A is essential for promoting centrosome duplication. The phosphorylation of *CEP250* induces the release of rootletin from the distal end of the centriole, which in turn leads to the centrosome disjunction [42]. Studies have confirmed that NDC80 and CEP250, in connection with NEK2, participated in the mechanism of some cancers*.* For example, NEK2 regulated G2/M phases through phosphorylation of *HEC1* [20], and HEC1 was crucial for faithful chromosome segregation and high-expression in most cancer cells. HEC1 is a homologous to NDC80 protein located at the budding yeast kinetochore during mitosis [20]. C-Nap1 (also known as CEP250) was demonstrated to be very important at the onset of mitosis and have an dynamic association with NEK2 [43]. It is suggested that NEK2 can phosphorylate multiple sites of C-Nap1 on the C-terminal domain (CTD), rather than a specific site, changing the overall charge of this domain and perturbing oligomerization and centrosome localization. These studies showed the strong connection between NDC80, CEP250 and NEK2.

MAD1L1, a component of the mitotic spindle-assembly checkpoint, belongs to MAD (mitotic arrest deficiency) family. MAD1L1 may play a pathogenic role in various types of human cancer [44] including HCC [45]. MAD1 interacts with NEK2A via a leucine zipper-containing domain located at the C terminus of *MAD1 *in vitro and in vivo [17]. *MAPK1* encodes a member of the MAP kinase family. MAP kinases, also known as extracellular signal-regulated kinases (ERKs), act as an integration point for multiple biochemical signals, and are involved in a wide variety of cellular processes such as proliferation, differentiation, transcription regulation and development. Studies have demonstrated that MAPK signaling pathway plays an important role in HCC [46, 47]. NEK2 may regulate proliferation, apoptosis, and other biological behaviors of HCC via MAPK signal pathway [48]. *PPP1CA* encodes protein phosphatase 1 (PP1) that is essential for cell division and participates in the regulation of glycogen metabolism, muscle contractility and protein synthesis. Few studies have presented its role in HCC currently. However, PP1 alpha was identified as a likely physiological antagonist of NEK2 [49]. Therefore, PP1 encoded by *PPP1CA* may influence the function of NEK2. The results from DAVID and KOBAS indicated that MAPK1 and PPP1CA were enriched in most pathways whereas NEK2 did not enriched in most pathways. This may be related to the fact that data about the roles of NEK2 in these pathways are still rare at present. STRING database verified that there was weak interaction between MAPK1, PPP1CA and NEK2. NEK2 might play its roles through interacting with MAPK1 and PPP1CA and result in alterations in biological pathways although further studies are needed to clarify this possibility. PPP1R2 belongs to PPP1R (protein phosphatase 1 regulatory) family, which participates in the inhibition of protein serine/threonine phosphatase. Evidence pertaining the role of PPP1R2 in HCC is scarce, but PPP1R42 (protein phosphatase 1, regulatory subunit 42) depletion may reduce the activity of PP1, leading to activation of NEK2 [50]. Therefore, PPP1R family may assist NEK2 in its role in HCC. NEK11 is a member of the never in mitosis gene A family of kinases, appears to play roles in DNA replication and response to genotoxic stress, and may function with NEK2A in the S-phase checkpoint [22]. Its role in HCC has not been clarified. To sum up, MAD1L1, MAPK1, PPP1CA, PPP1R2, and NEK11 all have something to do with NEK2. Certainly, the detailed roles of these proteins in HCC need to be verified.

In this study, there were some contradictory results. For example, the molecular function of GO terms in STRING database was enriched by the interesting proteins, but the DAVID database did not show similar result. The number of enriched proteins in the pathways of STRING database was higher than in those of DAVID and KOBAS 3.0 databases. Also, MAPK1 and PPP1CA were enriched in most KEGG pathways, but the interesting proteins (NEK2, NDC80 and CEP250) did not enriched in KEGG pathways. The inconsistencies of pathways among different analyses in our study might be related to the differences of data sources and analyzing focus in different databases. STRING and Reactome databases contain more data from different sources than DAVID and KOBAS 3.0 databases. Reactome database focuses on combining signaling, metabolic molecules and biological pathways into a vivid map. The core unit of the Reactome data model is the reaction. The functions of DAVID and KOBAS 3.0 databases concentrate on annotation and functional gene set enrichment. Differences between databases may lead to different results. However, there was a consistency between the different databases. Namely, cell cycle pathway was presented in all the analyses. Therefore, NEK2 and its interacting proteins might be implicated in the pathogenesis of HCC through cell cycle pathway, especially mitotic prometaphase pathway, a subset of cell cycle. NEK2, NDC80, CEP250 and MAD1L1 were the proteins enriched in mitotic prometaphase pathway. These results support that NEK2, NDC80 and CEP250 may coordinately play important roles in influencing HCC development. In univariate Cox regression model, NEK2, NDC80, and CEP250 were all significantly associated with HCC patient’s survival. In multivariate Cox regression model, NDC80 was an independent predictor for HCC patients’ survival, but NEK2 and CEP250 were not independent predictors. We speculate that the function of NEK2 was influenced by NDC80. In univariate Cox regression model, the interrelationship exited between NEK2, NDC80 and CEP250, suggesting the influential role of NEK2 and CEP250 on the survival of HCC patients. In multivariate Cox regression model, the interrelationship between NEK2, NDC80 and CEP250 was diminished, and the influence of NEK2 and CEP250 on HCC patients’ survival disappeared. We speculate that NEK2 alone may play a weak role in HCC. Genesense analysis showed that NDC80 was the downstream protein of NEK2. Therefore, NEK2 might exert its effect on HCC mainly by influencing NDC80. CCNB1, CCNB2 and CDK1 were all proteins that participate in mitotic prometaphase pathway, and the mRNA expression of the three genes have been shown to have strong positive correlation with *NEK2* mRNA expression. Genesense website did not show the upstream and downstream relationship between CCNB1, CCNB2, CDK1 and NEK2, but the high correlation coefficient suggested that there must be some relationship between these proteins. NEK2, NDC80, and CEP250 may play some roles together with CCNB1, CCNB2 and CDK1 in mitotic prometaphase pathway. The survival analysis outcome in our study was similar with the study by Li et al. [13]. The clinical covariates in the study of Li et al. could not be incorporated in our analysis because our study used the median value of *NEK2* mRNA expression while Li et al. used the cut-off point of *NEK2* mRNA expression defined by the receiver operating characteristic curve to categorize the HCC patients to high or low expression groups. However, the conclusions of the two studies reach a consensus, that is, the prognosis of HCC patients with high *NEK2* mRNA expression was poorer than those with low expression.

Small molecule therapies targeting NEK2 and NDC80 in different tumors have been reported. *NEK2* siRNA was found to inhibit tumor growth in a subcutaneous xenograft mouse model of pancreatic cancer, prolong the survival time in an intraperitoneal xenograft mouse model and efficiently prevent the progression of liver metastasis [51]. Combination of *NEK2* siRNA and chemotherapeutic agents may be effective treatment for colorectal cancer [52]. NDC80 might be a good targeting option in suppressing breast cancer tumor growth, and dual targeting of NEK2 and NDC80 might improve the prognosis [53]. Currently, there have been no studies on molecular therapy by targeting CEP250. Whether targeting NEK2, NDC80 or CEP250 alone or in various combinations may be therapeutically feasible for HCC still needs experimental studies in future.

## Conclusions

In summary, the systematic bioinformatics analyses in this study showed that NEK2 and its interacting proteins (MAD1L1, CEP250, MAPK1, NDC80, PPP1CA, PPP1R2, NEK11), especially NEK2 key interacting proteins (NDC80 and CEP250), are significantly involved in HCC. Mitotic prometaphase pathway, a subset of cell cycle pathway, might be the crucial pathway that NEK2, NDC80 and CEP250 are involved in the pathogenesis of HCC. There are strong interactions between NEK2, NDC80 and CEP250. The mRNA expressions of *NEK2*, *NDC80* and *CEP250* were significantly associated with HCC patients’ survival. Patients with high mRNA expression and genetic alterations of *NEK2*, *NDC80* and *CEP250* had poor prognosis. These findings provide evidence for the potential application of NEK2 as well as NDC80 and CEP250 as biomarkers for HCC diagnosis and HCC patient prognosis and as targets for HCC therapy.

## Supplementary information

**Additional file 1**. ** Figure S1**: Flow chart of the study and the sample numbers for each data mining part.

**Additional file 2**. ** Figure S2**: The pathways that NEK2 and its interacting proteins participate in by Reactome database analysis. The red lines in the figure fireworks present the pathways that NEK2 and its interacting proteins may be involved in, and the blue lines present the pathways that NEK2 and its interacting proteins may not be involved in.

**Additional file 3**. ** Figure S3**: The details of mitotic prometaphase pathway. The purple boxes represent the main biological processes that NEK2 and its interacting proteins may involve in.

**Additional file 4**. ** Figure S4**: The correlation of mRNA expression between NDC80, CEP250, CCNB1, CCNB2, CDK1 and NEK2 from TCGA database.** A**. NEK2 vs. NDC80;** B**. NEK2 vs. CEP250;** C**. NEK2 vs. CCNB1;** D**. NEK2 vs. CCNB2;** E**. NEK2 vs. CDK1.

**Additional file 5**. **Figure S5**: The correlation of mRNA expression from cBioPortal database. A. NEK2 vs. NDC80; B. NEK2 vs. CEP250.

**Additional file 6**. ** Table S1**: The characteristics of 373 HCC patients from TCGA database.

## Data Availability

The datasets used and analyzed in this study are available from the corresponding websites of the databases, including UALCAN: https://ualcan.path.uab.edu/cgi-bin/TCGAExResultNew2.pl?genenam=NEK2&ctype=LIHC; GeneSense: https://www.biomedsense.org/g_interloop2.php?g=NEK2; STRING: https://string-db.org/cgi/network.pl?taskId=sYn3BXkN9fGj; DAVID: https://david-d.ncifcrf.gov/ (The analysis criteria in the method section of our article need to be re-entered to see our results); KOBAS3.0: https://kobas.cbi.pku.edu.cn/kobas3/genelist/ (The analysis criteria in the method section of our article need to be re-entered to see our results); Reactome: https://reactome.org/PathwayBrowser/#/DTAB=AN&ANALYSIS=MjAyMDA2MTgwMDA0NTlfNjc5&FILTER=resource:UNIPROT; Oncomine: https://www.oncomine.org/resource/login.html (Registration is needed to login into the database) and cBioPortal: https://www.cbioportal.org/results/oncoprint?Action=Submit&RPPA_SCORE_THRESHOLD=2.0&Z_SCORE_THRESHOLD=2.0&cancer_study_list=lihc_tcga&case_set_id=lihc_tcga_cnaseq&data_priority=0&gene_list=NEK2%252C%2520NDC80%252C%2520CEP250&geneset_list=%20&genetic_profile_ids_PROFILE_COPY_NUMBER_ALTERATION=lihc_tcga_gistic&genetic_profile_ids_PROFILE_MUTATION_EXTENDED=lihc_tcga_mutations&profileFilter=0&tab_index=tab_visualize&genetic_profile_ids_PROFILE_MRNA_EXPRESSION=lihc_tcga_rna_seq_v2_mrna_median_Zscores. The results were often aggregated from multiple databases and analyzed online without the need to download the data. The data of the 373 HCC patients from TCGA database were downloaded from https://portal.gdc.cancer.gov/repository?cases_offset=200&facetTab=cases&filters=%7B%22op%22%3A%22and%22%2C%22content%22%3A%5B%7B%22op%22%3A%22in%22%2C%22content%22%3A%7B%22field%22%3A%22cases.primary_site%22%2C%22value%22%3A%5B%22liver%20and%20intrahepatic%20bile%20ducts%22%5D%7D%7D%2C%7B%22op%22%3A%22in%22%2C%22content%22%3A%7B%22field%22%3A%22cases.project.project_id%22%2C%22value%22%3A%5B%22TCGA-LIHC%22%5D%7D%7D%5D%7D&searchTableTab=cases. All data generated during this study are included in this published article.

## Abbreviations

AFP: Alpha-fetoprotein
BP: Biological process
CC: Cellular component
CEP250: Centrosomal protein 250 kDa
CNA: Copy number alteration
CTD: C-terminal domain
DAVID: The Database for Annotation, Visualization and Integrated Discovery
ERK: Extracellular signal-regulated kinase
FDR: False discovery rate
GISTIC: Genomic identification of significant targets in cancer
GO: Gene Ontology
HCC: Hepatocellular carcinoma
KEGG: Kyoto Encyclopedia of Genes and Genomes
KOBAS: KEGG Orthology Based Annotation System
MAD: Mitotic arrest deficiency
MAD1L1: MAD1 mitotic arrest deficient-like 1 (yeast)
MAPK1: Mitogen-activated protein kinase 1
MF: Molecular function
MMP-2: Matrix metalloproteinases-2
NDC80: Nuclear division cycle 80
NEK2: Never in mitosis gene A-related kinase 2
NEK11: NIMA (never in mitosis gene a)-related kinase 11
NuMA: Nuclear mitotic apparatus protein
PP1: Protein phosphatase 1
PPI: Protein–protein interaction
PPP1CA: Protein phosphatase 1 catalytic subunit alpha
PPP1R: Protein phosphatase 1 regulatory
PPP1R2: Protein phosphatase 1, regulatory (inhibitor) subunit 2
PPP1R42: Protein phosphatase 1, regulatory subunit 42
SD: Standard deviation
STRING: Search Tool for the Retrieval of Interacting Genes/Proteins
TCGA: The Cancer Genome Atlas
UALCAN: University of Alabama Cancer Database
