# Supplementary figures and images for: Involvement of NEK2 and its interaction with NDC80 and CEP250 in hepatocellular carcinoma
Source: BMC Med Genomics. 2020 Oct 27;13:158. doi: 10.1186/s12920-020-00812-y (PMC7590453; doi:10.1186/s12920-020-00812-y)

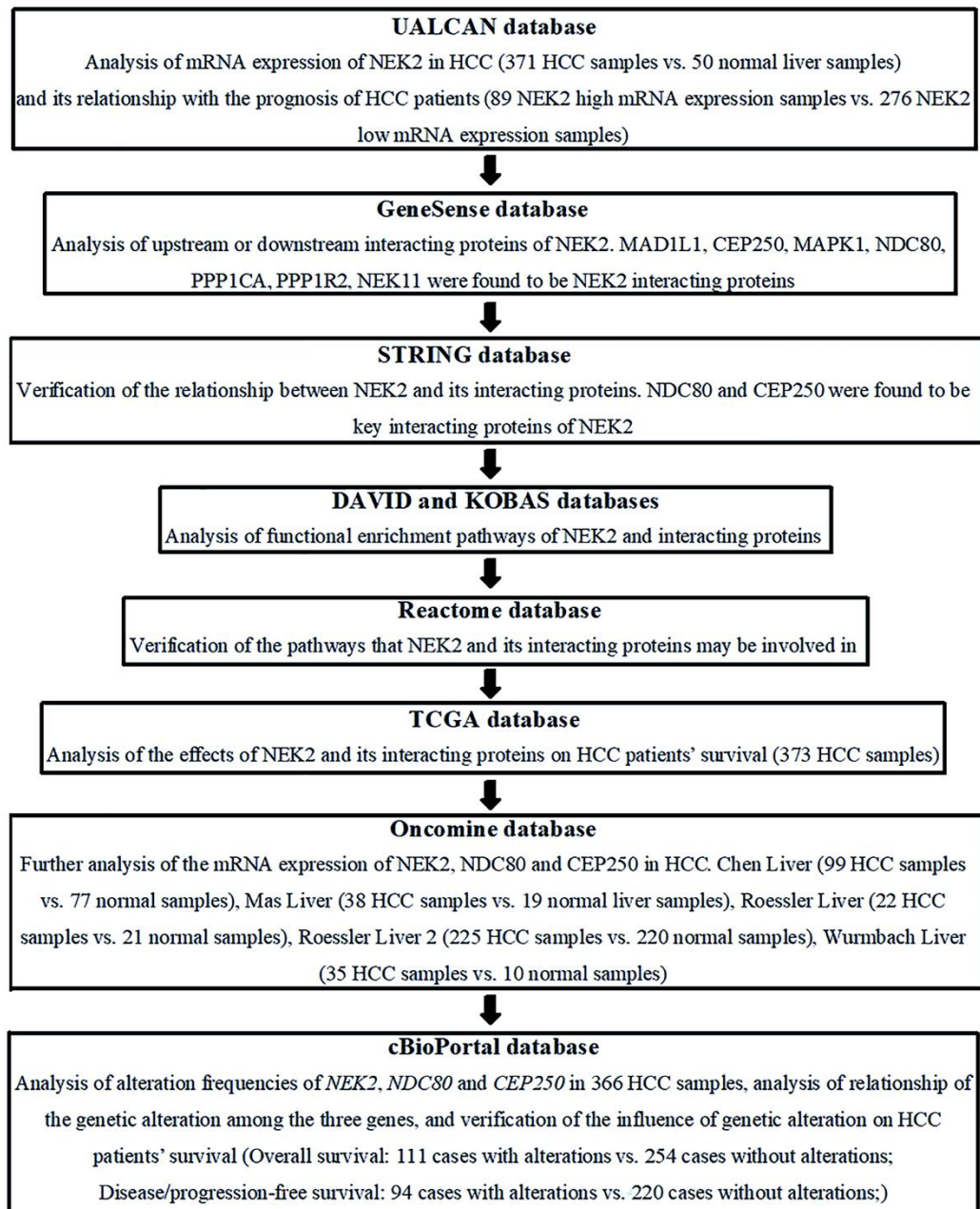

Figure S1. Flow chart of the study and the sample numbers for each data mining part.

Supplement: Supplementary file 1 — Additional file 1. Figure S1: Flow chart of the study and the sample numbers for each data mining part. [file 12920_2020_812_MOESM1_ESM.pdf]

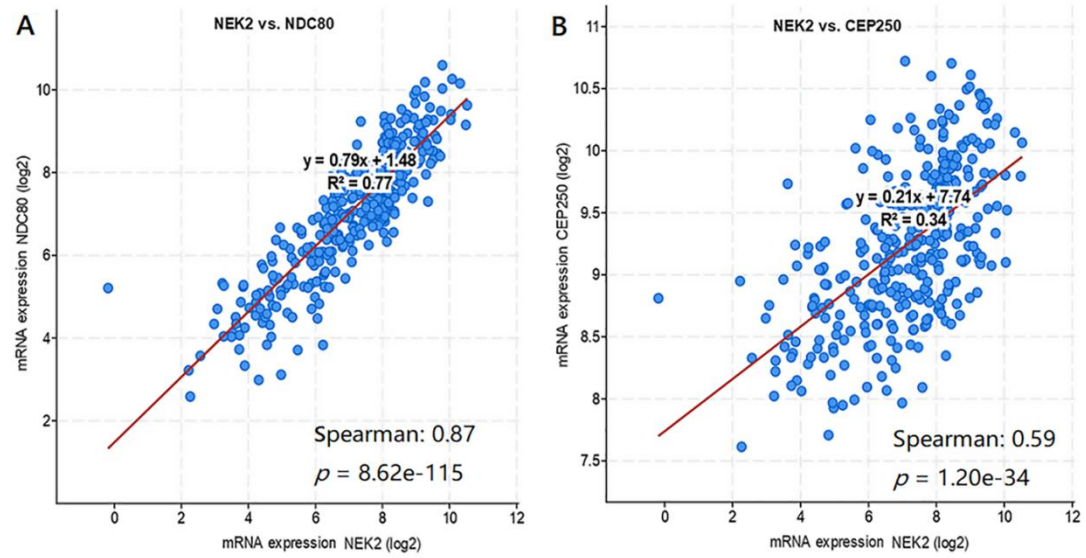

Figure S5. The correlation of mRNA expression from cBioPortal database. **A.** NEK2 vs. NDC80; **B.** NEK2 vs. CEP250.

Supplement: Supplementary file 5 — Additional file 5. Figure S5: The correlation of mRNA expression from cBioPortal database. A. NEK2 vs. NDC80; B. NEK2 vs. CEP250. [file 12920_2020_812_MOESM5_ESM.pdf]
