# Supplementary material for: Involvement of NEK2 and its interaction with NDC80 and CEP250 in hepatocellular carcinoma
Source: BMC Med Genomics. 2020 Oct 27;13:158. doi: 10.1186/s12920-020-00812-y (PMC7590453; doi:10.1186/s12920-020-00812-y)
Supplement: Supplementary file 2 — Additional file 2. Figure S2: The pathways that NEK2 and its interacting proteins participate in by Reactome database analysis. The red lines in the figure fireworks present the pathways that NEK2 and its interacting proteins may be involved in, and the blue lines present the pathways that NEK2 and its interacting proteins may not be involved in. [file 12920_2020_812_MOESM2_ESM.pdf]

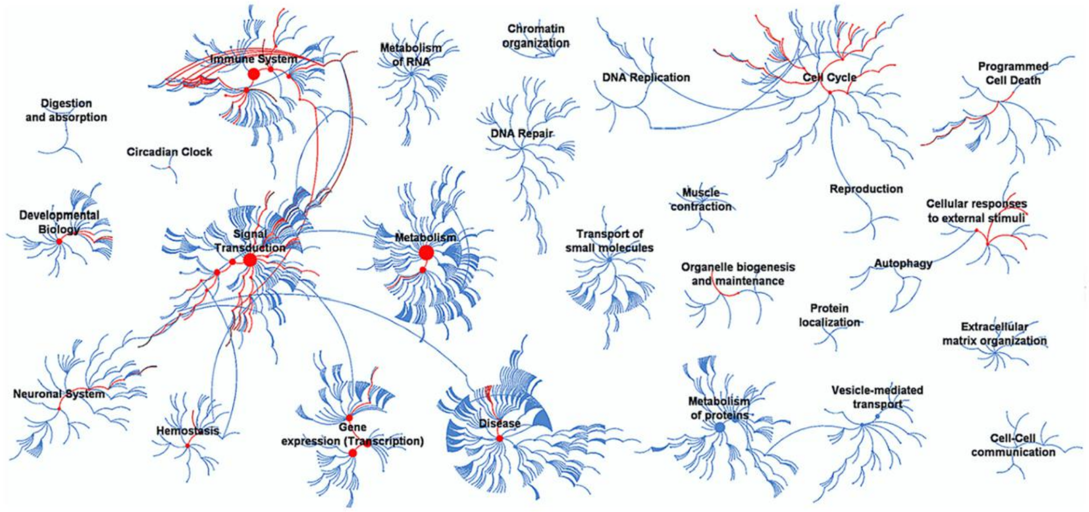

Figure S2. The pathways that NEK2 and its interacting proteins participate in by Reactome database analysis. The red lines in the figure fireworks present the pathways that NEK2 and its interacting proteins may be involved in, and the blue lines present the pathways that NEK2 and its interacting proteins may not be involved in.
