# Supplementary material for: Involvement of NEK2 and its interaction with NDC80 and CEP250 in hepatocellular carcinoma
Source: BMC Med Genomics. 2020 Oct 27;13:158. doi: 10.1186/s12920-020-00812-y (PMC7590453; doi:10.1186/s12920-020-00812-y)
Supplement: Supplementary file 3 — Additional file 3. Figure S3: The details of mitotic prometaphase pathway. The purple boxes represent the main biological processes that NEK2 and its interacting proteins may involve in. [file 12920_2020_812_MOESM3_ESM.pdf]

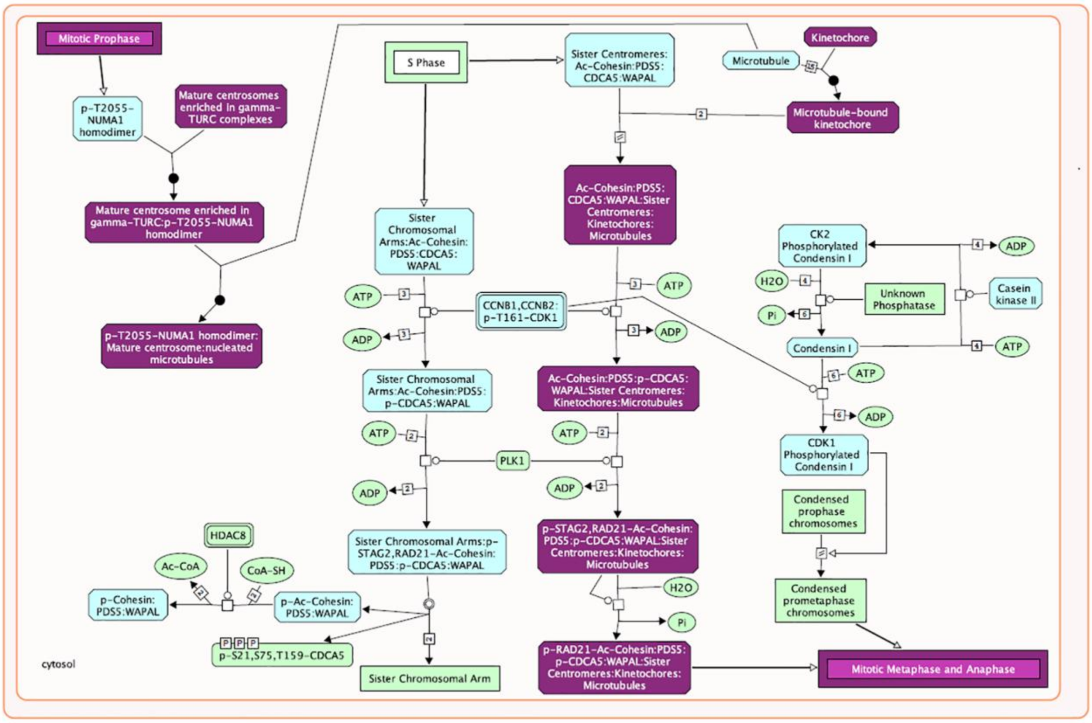

Figure S3. The details of mitotic prometaphase pathway. The purple boxes represent the main biological processes that NEK2 and its interacting proteins may involve in.
