# Supplementary material for: Involvement of NEK2 and its interaction with NDC80 and CEP250 in hepatocellular carcinoma
Source: BMC Med Genomics. 2020 Oct 27;13:158. doi: 10.1186/s12920-020-00812-y (PMC7590453; doi:10.1186/s12920-020-00812-y)
Supplement: Supplementary file 4 — Additional file 4. Figure S4: The correlation of mRNA expression between NDC80, CEP250, CCNB1, CCNB2, CDK1 and NEK2 from TCGA database. A. NEK2 vs. NDC80; B. NEK2 vs. CEP250; C. NEK2 vs. CCNB1; D. NEK2 vs. CCNB2; E. NEK2 vs. CDK1. [file 12920_2020_812_MOESM4_ESM.pdf]

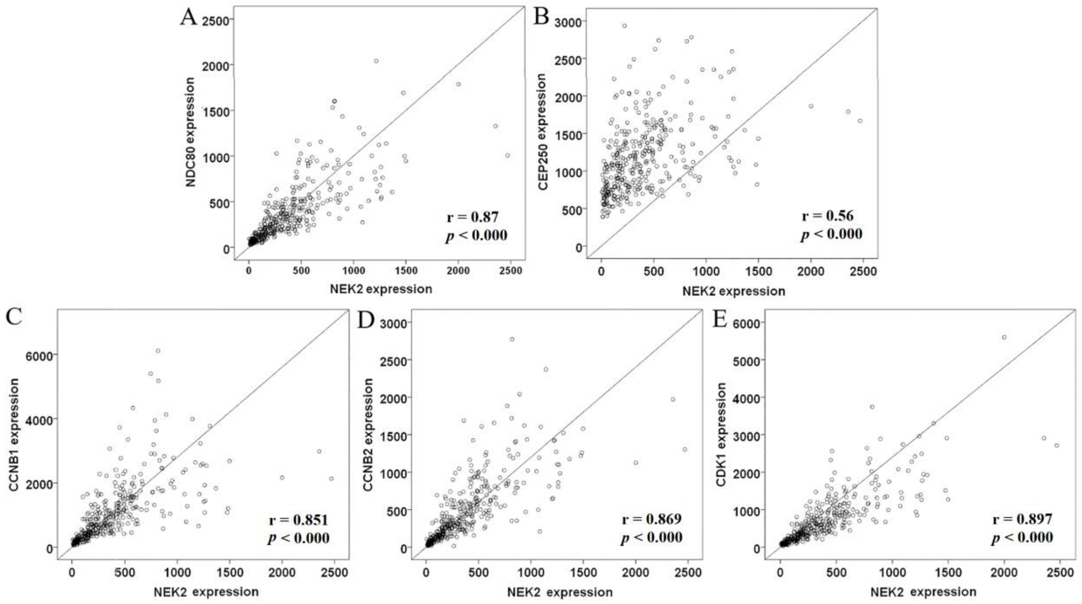

Figure S4. The correlation of mRNA expression between NDC80, CEP250, CCNB1, CCNB2, CDK1 and NEK2 from TCGA database. **A**. NEK2 vs. NDC80; **B**. NEK2 vs. CEP250; **C**. NEK2 vs. CCNB1; **D**. NEK2 vs. CCNB2; **E**. NEK2 vs. CDK1.
