# Supplementary material for: Involvement of NEK2 and its interaction with NDC80 and CEP250 in hepatocellular carcinoma
Source: BMC Med Genomics. 2020 Oct 27;13:158. doi: 10.1186/s12920-020-00812-y (PMC7590453; doi:10.1186/s12920-020-00812-y)
Supplement: Supplementary file 6 — Additional file 6. Table S1: The characteristics of 373 HCC patients from TCGA database. [file 12920_2020_812_MOESM6_ESM.doc]

| Characteristics | n | NEK2 | | NDC80 | | CEP250 | |
| --- | --- | --- | --- | --- | --- | --- | --- |
|  |  | mean rank | *p*-value | mean rank | *p*-value | mean rank | *p*-value |
| Age (year) | 373 |  | 0.018 |  | 0.016 |  | 0.777 |
| <60 | 169 | 201.55 |  | 201.80 |  | 185.26 |  |
| ≥60 | 204 | 174.95 |  | 174.74 |  | 188.44 |  |
| Gender | 373 |  | 0.383 |  | 0.926 |  | 0.074 |
| Female | 121 | 194.03 |  | 187.75 |  | 201.41 |  |
| Male | 252 | 183.62 |  | 186.64 |  | 180.08 |  |
| Family history of HCC | 321 |  | 0.003 |  | 0.02 |  | 0.021 |
| No | 209 | 172.46 |  | 169.82 |  | 169.73 |  |
| Yes | 112 | 139.61 |  | 144.54 |  | 144.71 |  |
| Recurrence | 289 |  | 0.009 |  | 0.013 |  | 0.002 |
| No | 148 | 132.40 |  | 133.11 |  | 129.87 |  |
| Yes | 141 | 158.23 |  | 157.48 |  | 160.88 |  |
| Weight(kg) | 345 |  | 0.001 |  | 0.001 |  | <0.001 |
| <73 | 206 | 187.42 |  | 188.05 |  | 190.58 |  |
| ≥73 | 139 | 151.63 |  | 150.69 |  | 146.94 |  |
| Platelet count (×10^9^/L) | 274 |  | 0.538 |  | 0.848 |  | 0.604 |
| >218 | 108 | 141.16 |  | 138.64 |  | 140.57 |  |
| ≤218 | 166 | 135.12 |  | 136.76 |  | 135.50 |  |
| Child-Pugh score | 240 |  | 0.466 |  | 0.515 |  | 0.603 |
| A | 218 | 120.94 |  | 122.04 |  | 121.05 |  |
| B | 21 | 120.00 |  | 106.57 |  | 118.10 |  |
| C | 1 | 35.00 |  | 78.00 |  | 52.00 |  |
| Histological grade | 368 |  | <0.001 |  | <0.001 |  | <0.001 |
| G1 | 55 | 132.44 |  | 135.56 |  | 151.38 |  |
| G2 | 178 | 168.70 |  | 169.59 |  | 165.83 |  |
| G3 | 123 | 225.21 |  | 221.35 |  | 223.56 |  |
| G4 | 12 | 240.25 |  | 252.25 |  | 212.92 |  |
| Pathologic stage | 349 |  | 0.001 |  | <0.001 |  | 0.004 |
| StageⅠ | 174 | 155.57 |  | 155.24 |  | 155.76 |  |
| StageⅡ | 86 | 187.74 |  | 194.14 |  | 189.60 |  |
| Stage Ⅲ | 84 | 205.17 |  | 200.98 |  | 200.51 |  |
| Stage Ⅳ | 5 | 125.00 |  | 97.20 |  | 164.80 |  |
| Primary tumor range | 370 |  | <0.001 |  | 0.001 |  | 0.094 |
| T1 | 183 | 162.32 |  | 162.71 |  | 165.04 |  |
| T2 | 94 | 199.93 |  | 205.79 |  | 201.12 |  |
| T3 | 80 | 213.75 |  | 205.05 |  | 209.89 |  |
| T4 | 13 | 233.69 |  | 239.31 |  | 210.54 |  |
| Survival time (year) | 373 |  | 0.002 |  | 0.002 |  | 0.094 |
| >2 | 143 | 164.66 |  | 165.41 |  | 175.15 |  |
| ≤2 | 230 | 200.89 |  | 200.43 |  | 194.37 |  |

**Table S1**. The characteristics of 373 HCC patients from TCGA database.
